# Comfort level discussion for prosthetic sockets with different fabricating processing conditions

**DOI:** 10.1186/s12938-018-0577-2

**Published:** 2018-11-06

**Authors:** Cheung-Hwa Hsu, Chao-Hui Ou, Wei-Lun Hong, Yu-Han Gao

**Affiliations:** Department of Mold and Die Engineering, National Kaohsiung University of Science and Technology, Kaohsiung, Taiwan, ROC

**Keywords:** Prosthetic socket, Interface pressures, Rapid prototyping, Gait analysis

## Abstract

**Background:**

In the past, manufacture of prosthetic socket by using traditional handmade method not only consumed research time but also required a special assembly approach. Recently, reverse engineering and rapid prototype technology have grown up explosively, and thus, provide a choice to fabricate prosthetic socket.

**Methods:**

Application 3D computer aided design and manufacturing (computer-aided design/computer-aided engineering) tools approach the surface shape stump data is digitized and can be easily modified and reused. Collocation investigates gait parameters of prosthetic socket, and interface stress between stump and socket with different processing conditions. Meanwhile, questionnaire was utilized to survey satisfaction rating scale, comfort level, of subjects using this kind of artificial device.

**Results:**

The main outcome of current research including gait parameters, stress interface and satisfaction rating scale those would be an informative reference for further studies in design and manufacture as well as clinical applications of prosthetic sockets.

**Conclusions:**

This study found that, regardless of the method used for socket fabrication, most stress was concentrated in tibia end pressure-relief area. This caused discomfort in the area of tibia end to the participant wearing prosthesis. This discomfort was most evident in case when the prosthetic socket was fabricated using RE and RP.

## Background

Limb asymmetries in the physically disabled cause gait abnormalities, which is why the average gait speed in below-knee amputees (only 64 m/min) is lower than the normal gait speed of 91 m/min [1]. More comfortable walking can be achieved in unilateral amputees by increasing the prosthetic ankle angle [2]. Prostheses must be custom made for each amputee stump based on the individual needs of a patient. Much time should be allowed for adjusting a prosthetic socket and the alignment of prosthesis because the highest levels of patient satisfaction can be achieved only through multiple fitting trials, modifications, and adjustments. Traditionally, prosthetic sockets were produced via a manual and complicated manufacturing process conducted by experienced and technically skilled prosthesis. Computer software and hardware advances brought about a fast development of 3D computer aided design (CAD) and manufacturing (CAM) tools [3]. This study applied 3D CAD, reverse engineering (RE), and rapid prototyping (RP) techniques to develop a new technology to manufacture prosthetic sockets and avoid the inconveniences of the traditional handmade method. Gait analysis data of patients was recorded using a motion analysis system. Knee joint stresses and moments were computed through inverse dynamics. Indentation tests of soft tissues were used to measure pressure discomfort and pain thresholds. A special scale was developed for pain assessment such that combined a numeric rating scale (NRS) and visual analogue scale (VAS). In the 11-point NRS, 0 and 10 points represented the lowest and highest comfort levels for the prosthetic sockets, respectively. The VAS used a straight line to represent pain tolerance levels, with the two ends of the line indicating opposite extremes of pain [4].

## Methods

**Fig. 1:**
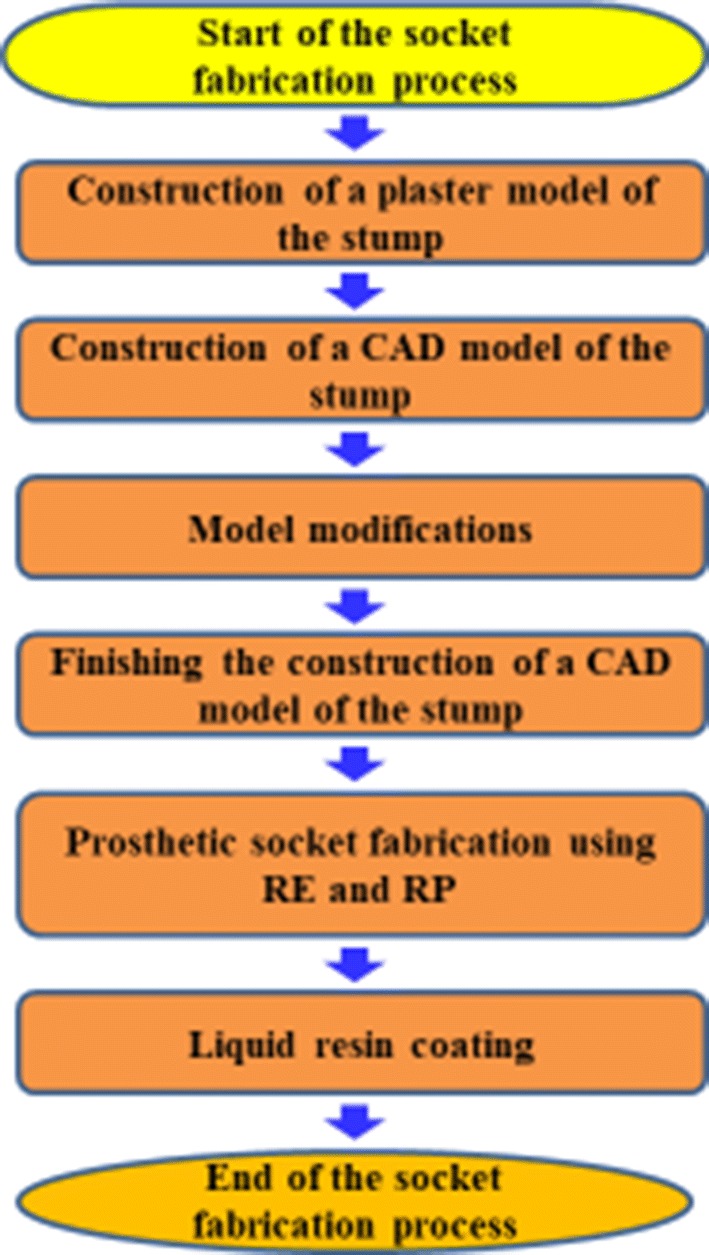
Prosthetic socket fabrication process

**Fig. 2:**
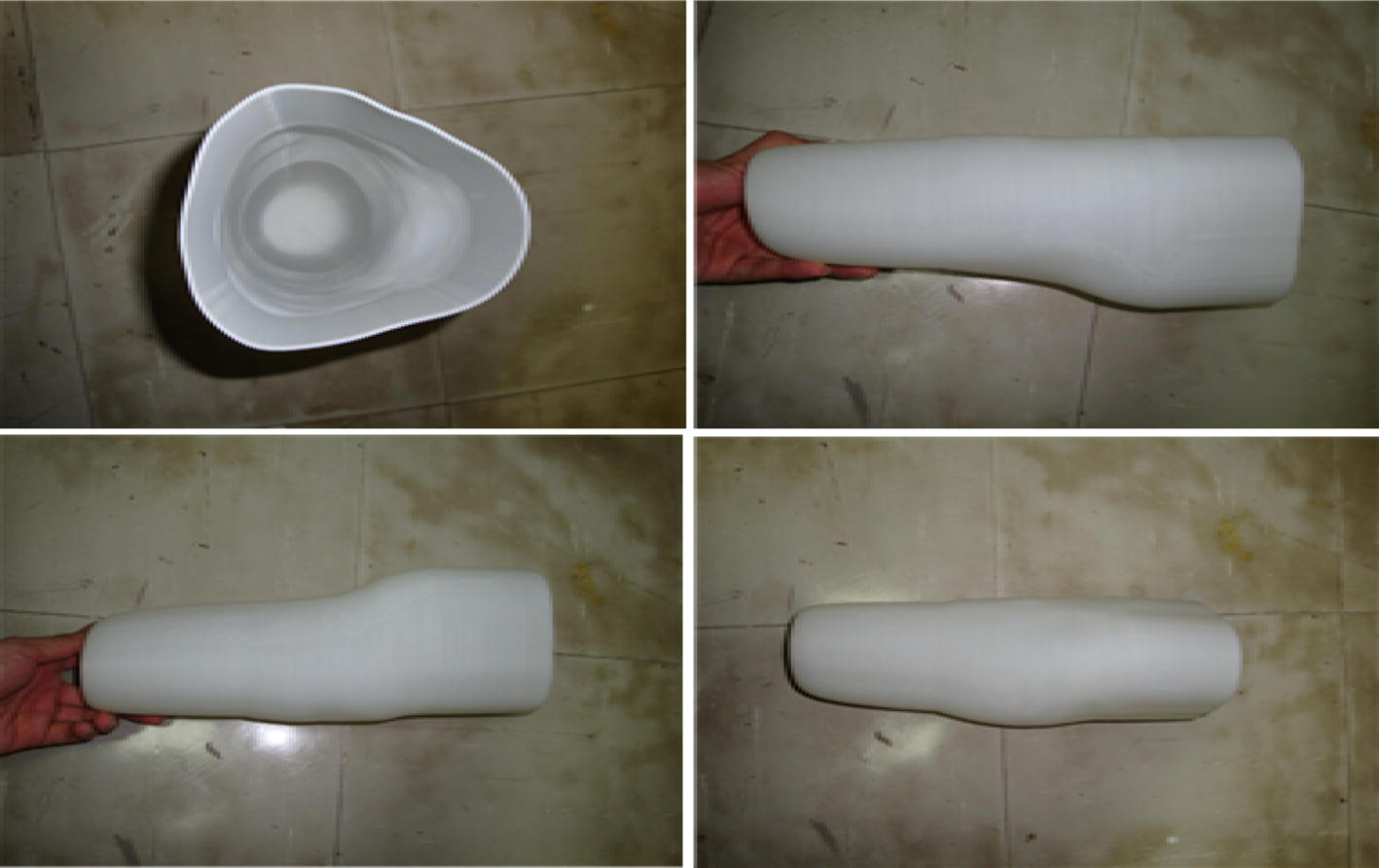
RP-fabricated prosthetic socket

**Fig. 3:**
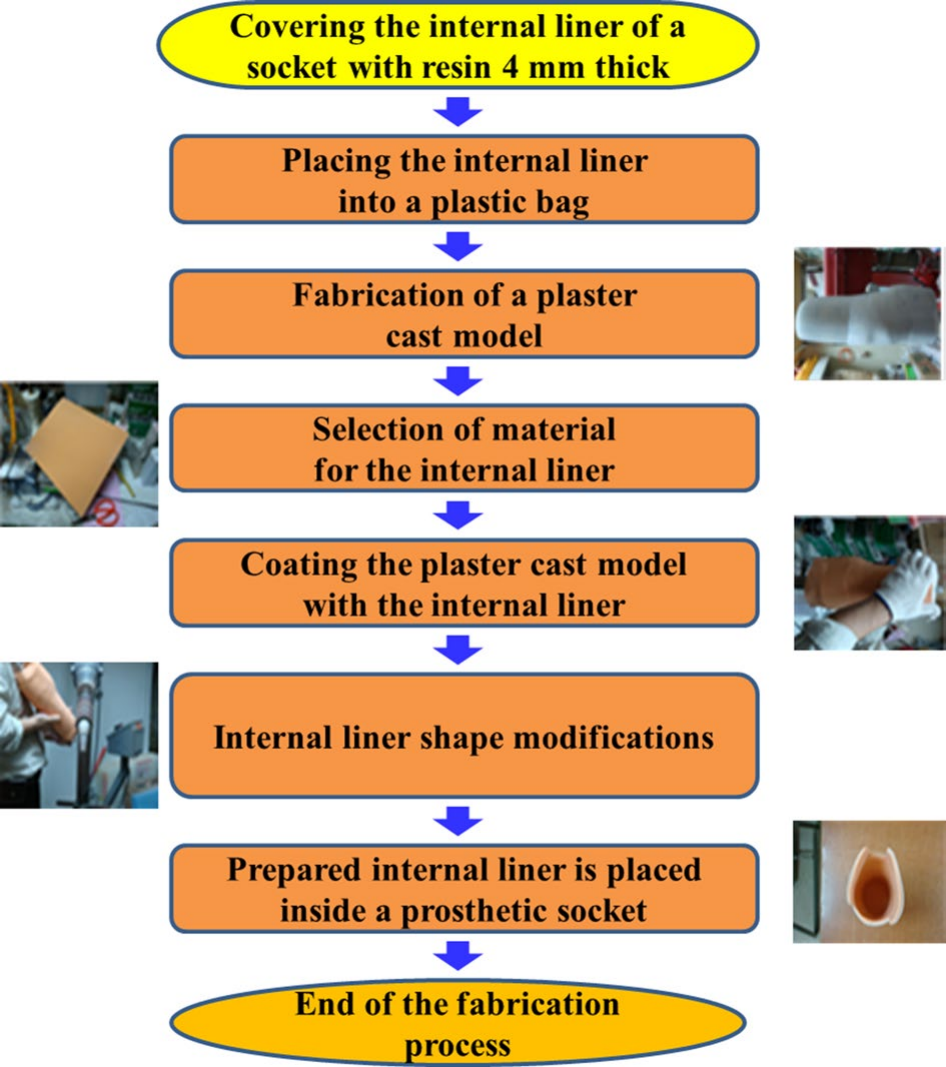
Internal liner fabrication process

Figure 1 shows the process of RP-based fabrication of prosthetic sockets. First, a plaster cast of the stump is taken by means of vacuum forming. Next, the stump model created using a computed tomography (CT) scan is imported into a drawing software to construct a 3D stump model and the stump surface is modified with respect to pressure-tolerant and pressure-relief areas. After these modifications and verification of the model by a specialist in prosthetics, it is processed in an RP machine and prosthetic sockets are fabricated (see Fig. 2) and coated with the liquid resin for greater strength and, thus, an amputee’s comfort. The internal liner serves as a stump–socket buffer. The inner surface of sockets is covered with a 4 mm thick material using RP and sockets are placed into plastic bags. Plaster is injected and, once dries and hard, the resulting plaster model is used as a model of the internal liner. Due to the use of rigid plastics in most prosthetic sockets, elastic and skin-like material should be selected for the internal liner, such as silica gel or polyurethane. The plaster cast model is then coated with the internal liner the material of which is heated to fit the curved surface. Finally, prosthesis modifies the liner shape to fit the plaster cast. The entire procedure of RP-based fabrication of a prosthetic socket is presented in Fig. 3. Insertion of the liner into the socket is the last step in such a manufacturing process.

## Results and discussion

Prosthetic sockets fabricated using the traditional handmade method and those fabricated using RE and RP were compared based on the differences in their gait analysis results. In gait analysis, a 3D space measurement system was applied to examine joint angle and time at each joint motion. Three types of parameters were studied, namely spatial and temporal parameters, kinematics parameters, and dynamics parameters. Instrumentation plans were designed according to the lower limb data and different positions of the patient were measured. Table 1 provides the participants body measurement data that was further used for dynamic analysis of their gait.

**Table 1:** Body measurement data of participants

Participants	Uni-lateral below-knee prosthesis users
Amputation side	Left
Sex	Male
Age	26
Height (cm)	175.0
Weight (kg)	84.1
Thigh length (cm)	Healthy = 48.7; amputated = 48.7
Thigh circumference (cm)	Healthy = 46.5; amputated = 46.5
Calf length (cm)	Healthy = 27.0; amputated = 19.2
Calf circumference (cm)	Healthy = 41.3; amputated = 33.5
Foot length (cm)	Healthy = 26.5; amputated = 25.0
Foot width (cm)	Healthy = 10.4; amputated = 9.5
Prosthesis weight (kg)	2.0

### Gait analysis

Spatial and temporal parameters (see Tables 2, 3) present spatial and temporal parameters of the prosthetic sockets manufactured with different methods. Their comparison showed that velocity and cadence were higher for the participant who wore the prosthetic socket fabricated with the traditional handmade method rather than that fabricated using RE and RP. This indicated that the handmade prosthetic socket better corresponded to the participant’s habits in terms of mobility and operation. In terms of stride length, larger values were observed when the prosthetic socket fabricated using RE and RP, rather than the traditional handmade method, was used. However, the former also demonstrated a greater difference in step length between an amputated and healthy limb, meaning that asymmetry between two limbs is a serious issue in the use of prosthetic sockets fabricated using RE and RP. The percentage of gait cycle spent in stance phase was found to be higher in the healthy side than the amputated side. This indicated that the participants tended to put a greater load on the healthy limb, while not willing to load the amputated limb, which was more evident in case of the prosthetic socket fabricated using RE and RP.

**Table 2:** Kinematics parameters of the healthy limb (mean SD)

Gait parameters	Prosthetic socket fabrication method	
	Traditional	RE and RP
Forward velocity (cm/s)	107.73 ± 5.34	98.25 ± 1.77
Cadence (steps/min)	89.11 ± 1.81	79.13 ± 0.87
Stride length (cm)	142.64 ± 4.67	149.29 ± 0.77
Step width (cm)	31.52 ± 1.94	30.78 ± 1.18
Step length (cm)	68.55 ± 2.43	68.11 ± 1.78
Stance phase (%)	64.29 ± 1.84	65.66 ± 0.10
Swing phase (%)	35.71 ± 1.84	34.34 ± 0.10

**Table 3:** Kinematics parameters of the amputated limb (mean SD)

Gait parameters	Prosthetic socket fabrication method	
	Traditional	RE and RP
Forward velocity (cm/s)	107.67 ± 4.29	96.91 ± 2.76
Cadence (steps/min)	90.35 ± 3.76	80.49 ± 1.80
Stride length (cm)	144.25 ± 2.15	145.88 ± 0.20
Step width (cm)	31.52 ± 1.94	30.78 ± 1.18
Step length (cm)	74.89 ± 2.95	79.47 ± 0.14
Stance phase (%)	63.44 ± 0.97	59.77 ± 0.34
Swing phase (%)	36.56 ± 0.97	40.23 ± 0.34

### Kinematics parameters

**Fig. 4:**
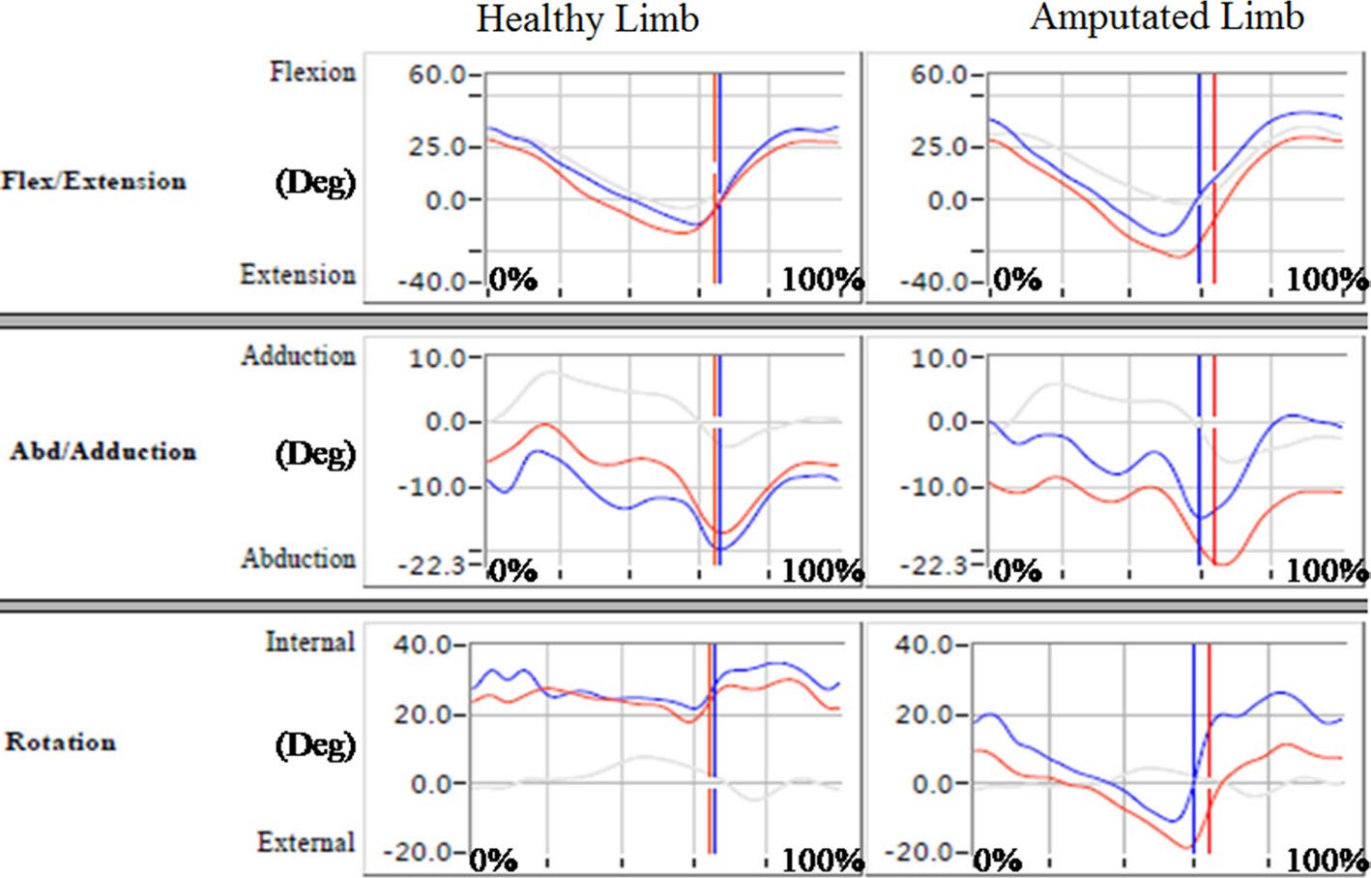
Hip joint angular changes in hip joints (red: traditional handmade method; blue: RE and RP; grey: normal value)

**Fig. 5:**
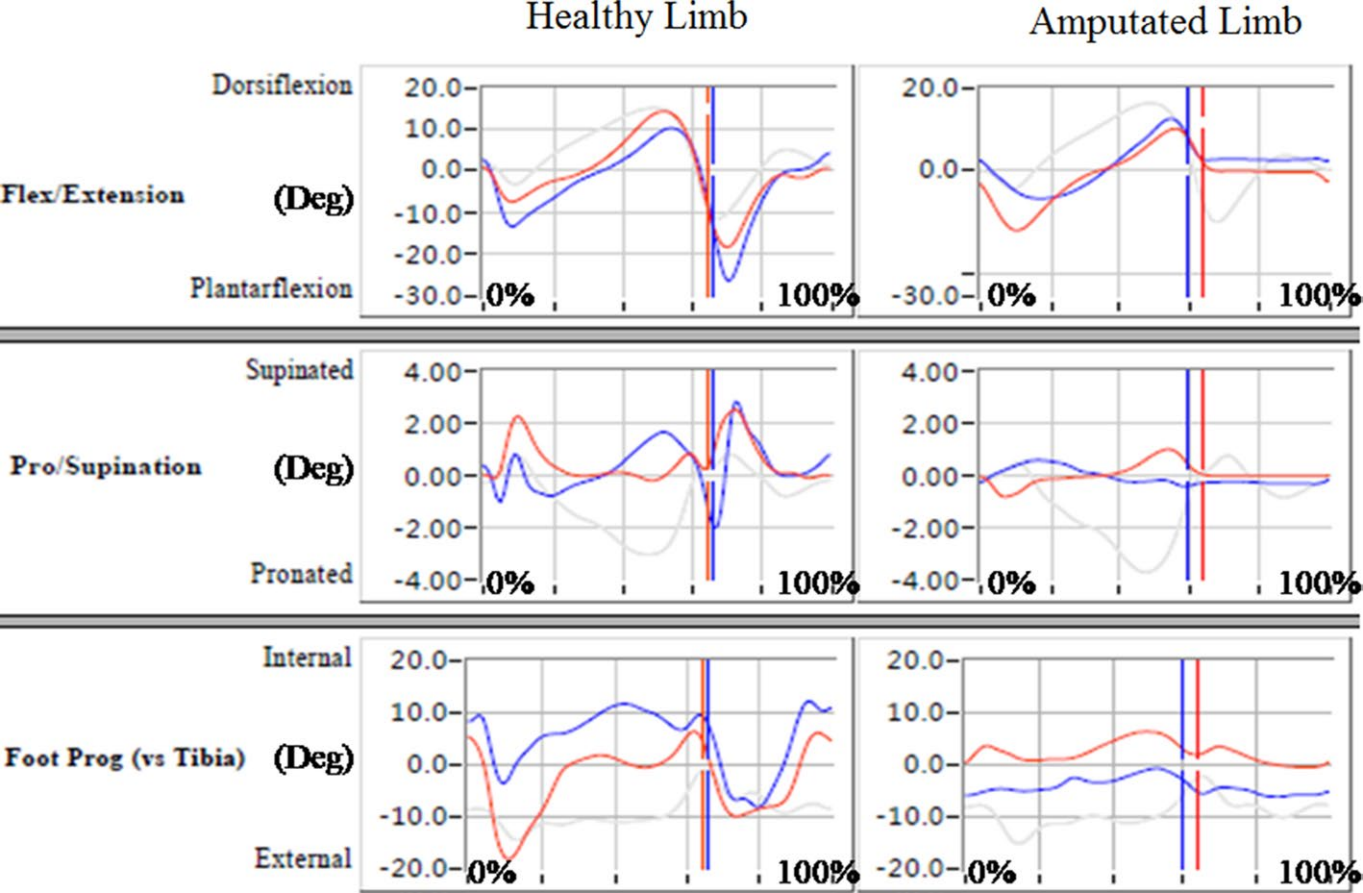
Knee joint angular changes in knee joints (red: traditional handmade method; blue: RE and RP; grey: normal value)

**Fig. 6:**
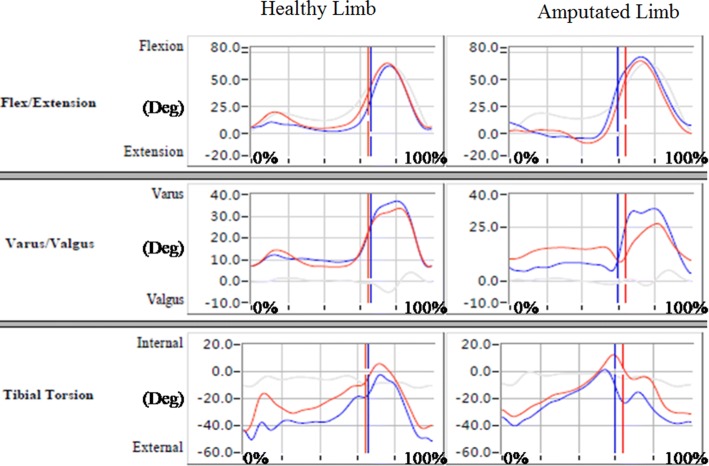
Ankle joint angular changes in ankle joints (red: traditional handmade method; blue: RE and RP; grey: normal value)

Figures 4, 5, and 6 show data on angular changes in the hip, knee, and ankle joints of the healthy and amputated limbs in sagittal, coronal, and transverse planes when wearing different prosthetic sockets. Angular changes in hip joints are presented in Fig. 4. In the sagittal and transverse planes, angular changes in the hip joint of the healthy limb did not differ greatly; in the coronal plane, when the prosthetic socket fabricated with the traditional handmade method was used, the hip joint angle of the healthy limb was larger than that for the prosthetic socket fabricated using RE and RP. With regard to the amputated limb, a relatively large extension angle in the sagittal plane in case of the prosthetic socket fabricated with the traditional handmade method indicated that it was better able to support the body weight and maintain balance than the prosthetic socket fabricated using RE and RP. Angular changes in knee joints are presented in Fig. 5. While no large differences were observed in the coronal and transverse planes, in the sagittal plane, healthy knee flexion during stance phase was more evident in the participant who wore the prosthetic socket fabricated with the traditional handmade method; it is inferred that low gait velocity decreased impact forces. Moreover, in the participant who wore the prosthetic socket fabricated using RE and RP, the amputated limb was not able to extend fully due to limited knee extension, lack of peak values, and socket locking inefficiency.

Angular changes in ankle joints are presented in Fig. 6. In the sagittal plane, greater ankle dorsiflexion range of motion and better performance in the healthy limb during the entire stance phase was observed when the prosthetic socket was used that was fabricated with the traditional handmade method. Due to the prosthetic socket fabricated using RE and RP being firmer in terms of adjustment to the amputated limb, the range of motion in the amputated limb in its case was not large. Depending on the socket fabrication method, peak values for the ankle joint angle considerably differed in the healthy limb in coronal and transverse planes and were higher in the amputated limb in the transverse plane when the prosthetic socket was fabricated with the traditional handmade method.

Comparison of spatial and temporal parameters and kinematics parameters showed that for greater step lengths in the healthy limb, the hip, knee, and ankle joint angles varied greatly in the coronal plane. In the sagittal plane, higher values for the amputated limb hip, knee, and ankle joint angles were observed when the prosthetic socket was fabricated using RE and RP. This is due to larger step length and shorter stance phase in case of such sockets.

### Dynamics analysis

**Fig. 7:**
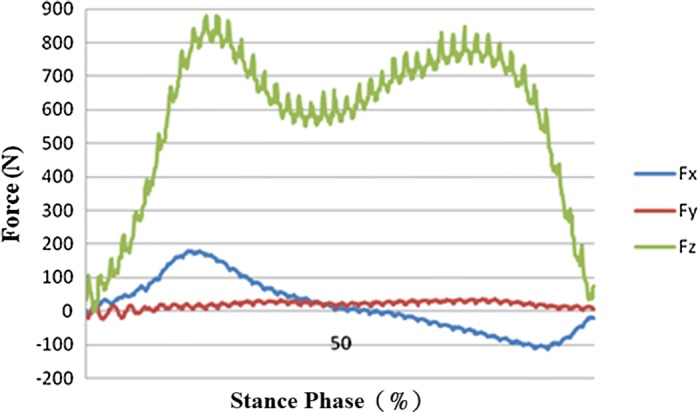
Force for the traditional handmade prosthetic socket

**Fig. 8:**
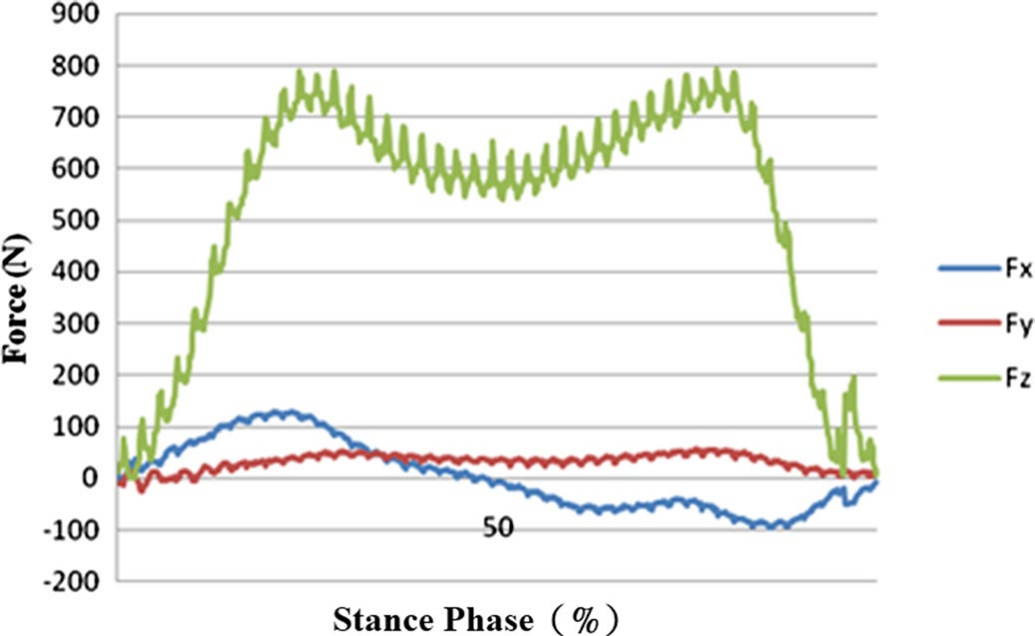
Force for the RE and RP prosthetic socket

Measurements of force in the amputated limb obtained using a force plate are given for prosthetic sockets fabricated using the traditional handmade method (see Fig. 7) and RE and RP (see Fig. 8). Two stress peaks occur in stance phases of 25% and 75%, particularly, when two-limb support changes into one-limb support with one limb leaving the ground and when one-limb support changes again into two-limb support with the leg stepping on the ground. Due to the lighter weight of the prosthetic socket fabricated using RE and RP, its force values were lower.

### Pressure pain measurement results

**Fig. 9:**
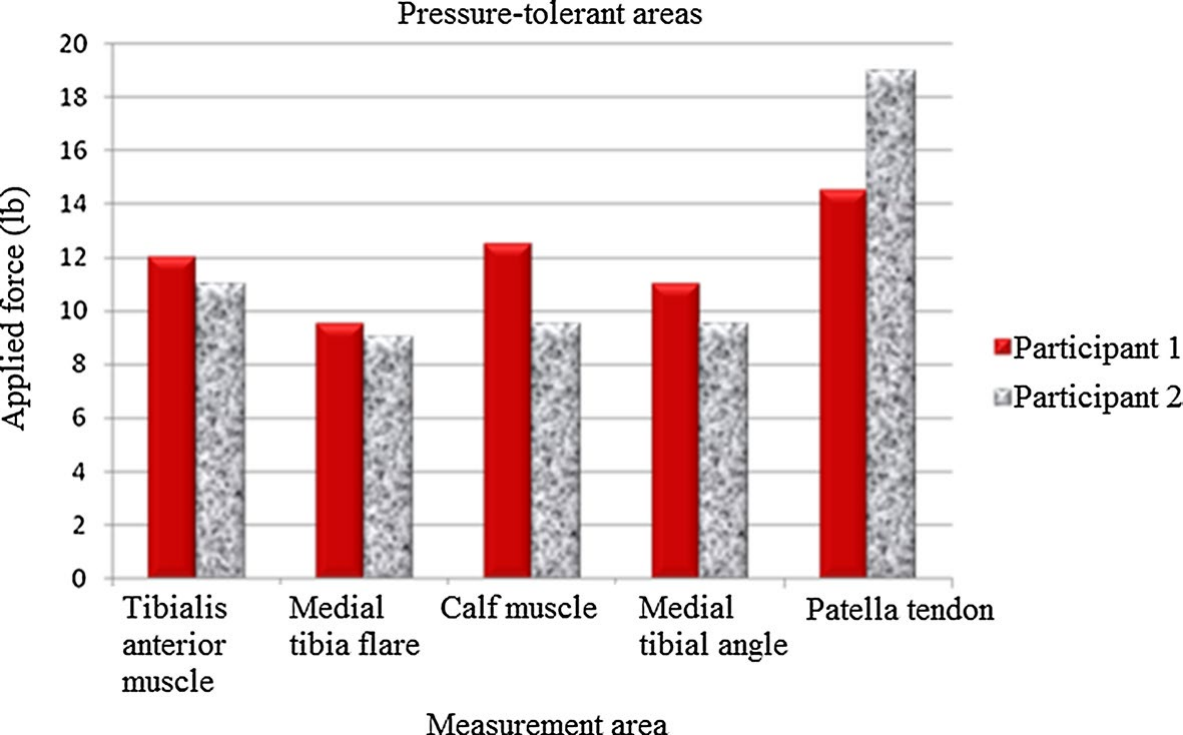
Comparison of pressure-tolerant areas

**Fig. 10:**
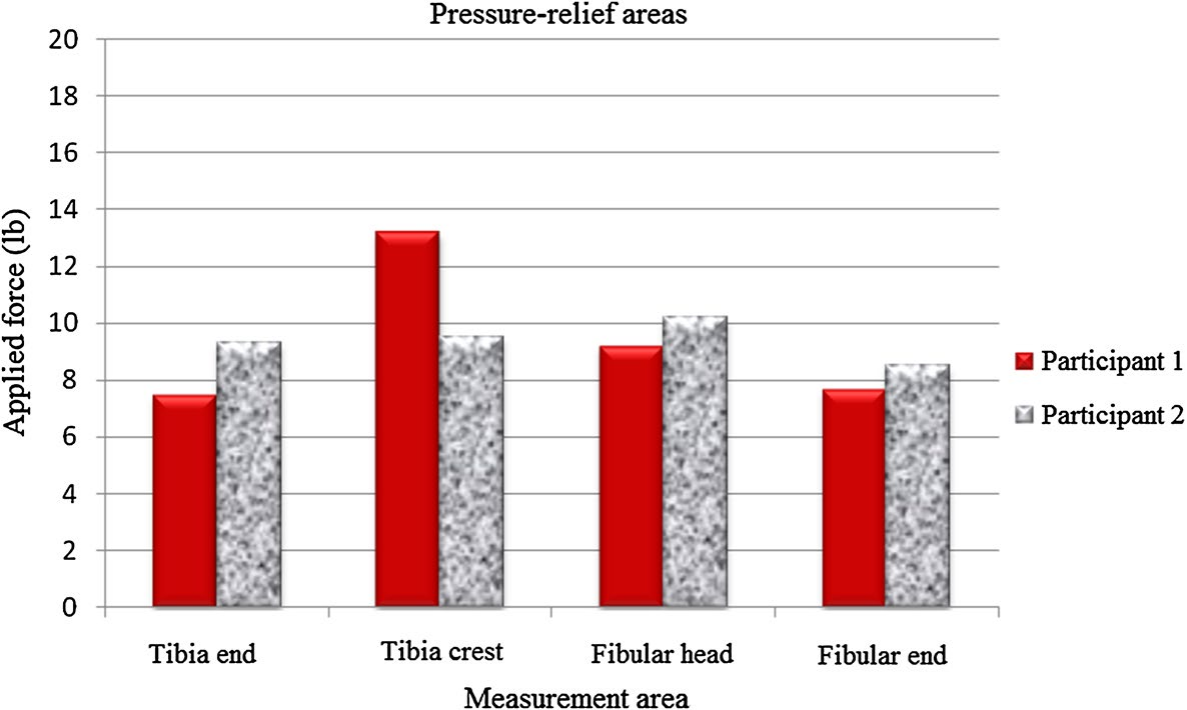
Comparison of pressure-relief areas

A pressure pain detector was used to measure pressure pain in the participants. A specialist made measurements in pressure-tolerant (patella tendon, anterior tibia muscle, medial tibia flare, medial tibia angle, and calf muscle) and pressure-relief (tibia crest, tibia end, fibular head, and fibular end) areas of the amputated limb to compare pressure pain tolerance in these areas. Empirical analysis results indicated that, on average, pressure pain tolerance in pressure-tolerant areas was higher than that in pressure-relief areas, with the highest and lowest values observed in the areas of patella tendon and fibular end, respectively, in both participants. Pressure pain measurement results for pressure-tolerant and pressure-relief areas are provided in Table 4, Figs. 9, and 10.

**Table 4:** Measurement results (unit N)

Measurement area	Case 1	Case 2
Pressure-tolerant areas (unit N)		
Tibias anterior muscle	12	11
Medial tibia flare	9.5	11
Calf muscle	12.5	9
Medial tibial angle	11	9.5
Patella tendon	14.5	19
Pressure-relief areas (unit N)		
Tibia end	7.5	9.3
Tibia crest	13.2	9.5
Fibular head	9.2	10.2
Fibular end	7.7	8.5

### Stump–socket interface stress results

**Fig. 11:**
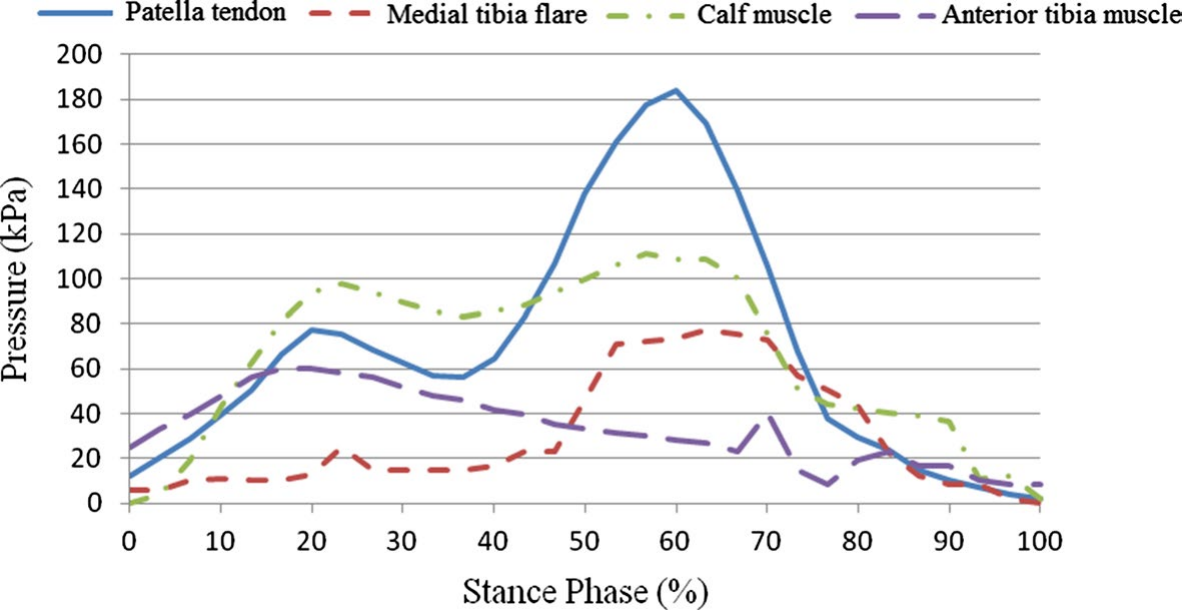
Interface stress of the prosthetic socket fabricated using the traditional handmade method in pressure-tolerant areas

**Fig. 12:**
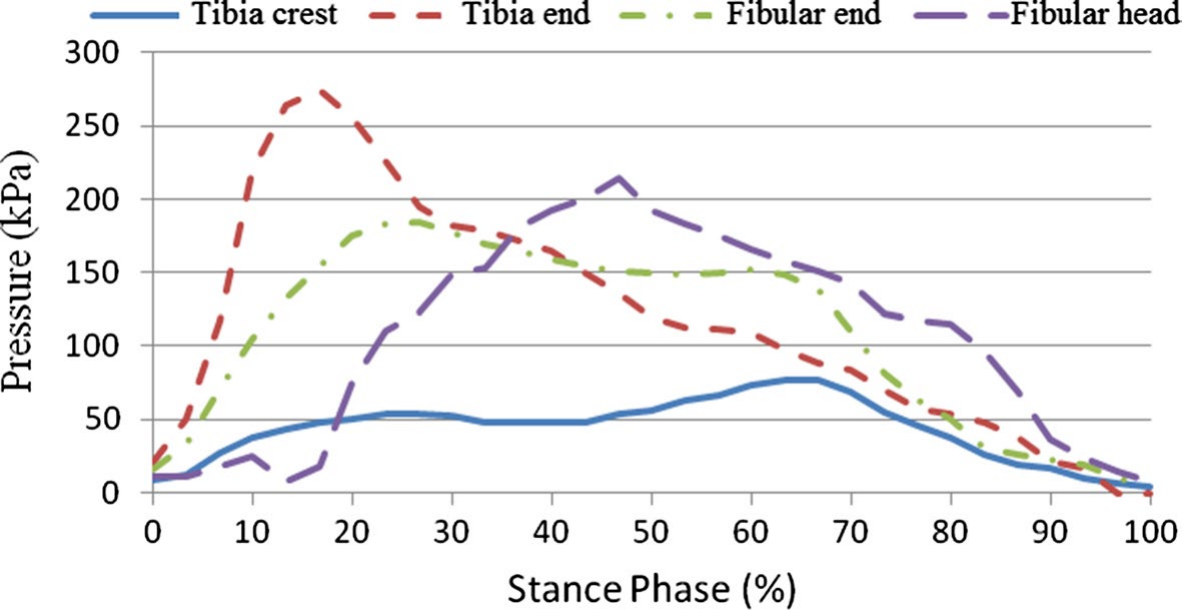
Interface stress of the prosthetic socket fabricated using the traditional handmade method in pressure-relief areas

**Fig. 13:**
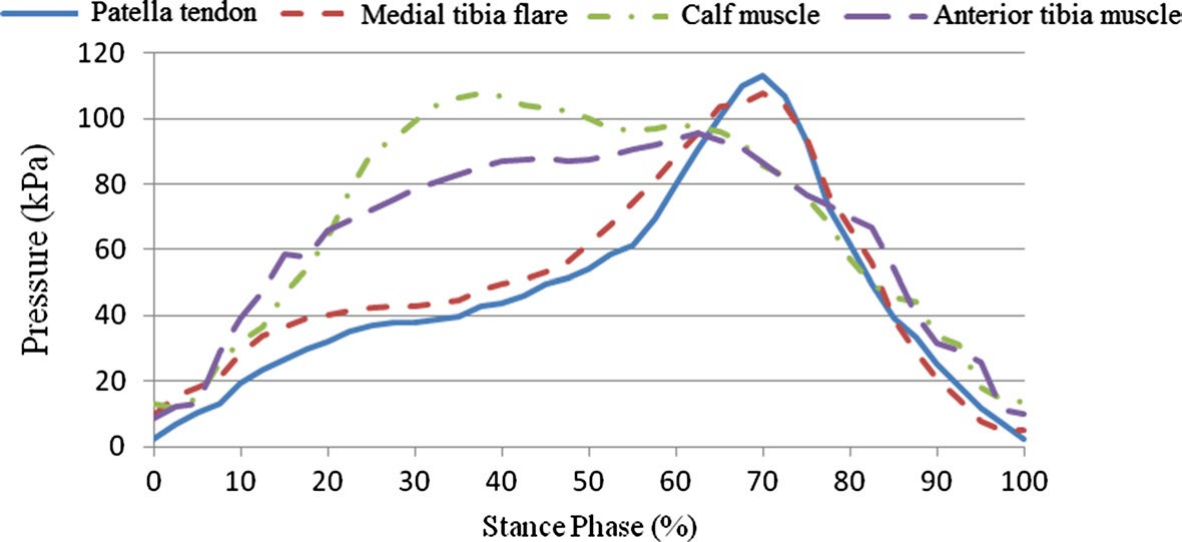
Interface stress of the prosthetic socket fabricated using RE and RP in pressure-tolerant areas

**Fig. 14:**
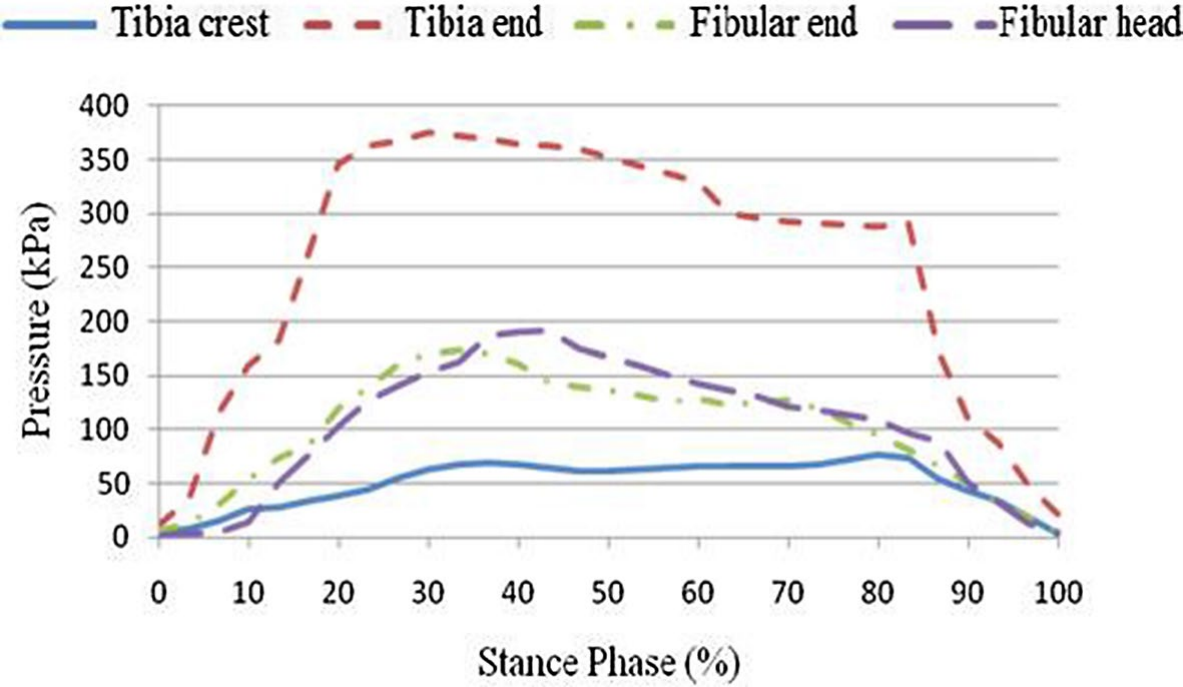
Interface stress of the prosthetic socket fabricated using RE and RP in pressure-relief areas

This study sought to examine stump–socket interface stress distribution in participants wearing prosthetic sockets fabricated using the traditional handmade method and RE and RP while walking at their normal gait velocity. Additionally, based on the dynamics data obtained from the force plate, boundary conditions were set for the quasi-static finite element model used for interface stress analysis, followed by comparison of empirical results. Interface stress was examined in a total of eight areas, including pressure-tolerant (patella tendon, medial tibia flare, calf muscle, and anterior tibia muscle) and pressure-relief (tibia crest, tibia end, fibular end, and fibular head) areas. Table 5 shows the peak values for stump–socket interface stress in different areas for the participants wearing the different prosthetic sockets. Figures 11 and 12 show the measurement results for the interface stress between a stump and the socket fabricated using the traditional handmade method in pressure-tolerant and pressure-relief areas, respectively. Figures 13 and 14 show the measurement results for the interface stress between a stump and the socket fabricated RE and RP in pressure-tolerant and pressure-relief areas, respectively.

**Table 5:** Interface stress of different prosthetic sockets (unit kPa)

Measurement area	Traditional handmade method	RE and RP
Patella tendon	183.85	113.02
Medial tibia flare	72.52	107.81
Calf muscle	110.93	107.77
Anterior tibia muscle	60.41	95.48
Tibia crest	77.08	77.67
Tibia end	274.79	372.10
Fibular end	183.85	173.14
Fibular head	214.65	192.12

As seen from Figs. 11, 12, 13, and 14, regardless of the socket fabrication method, the highest interface stress values were observed in the patella tendon among pressure-tolerant areas and the tibia end among pressure-relief areas. The interface stress in the patella tendon area was greater for the participant who wore the prosthetic socket fabricated with the traditional handmade method, meaning that such a socket received more loading than that which was fabricated using RE and RP, thus, prolonging the stance phase of the amputated limb. The interface stress in the medial tibia flare area was smaller for the participant who wore the prosthetic socket fabricated using the traditional handmade method, meaning that such a socket was larger than that which was fabricated using RE and RP, thus, imposing less stress on the enclosed stump. No substantial difference was found in interface stress values in the areas of the calf muscle and anterior tibia muscle for the two participants. In the fibular end area, stump–socket interface stress was larger for the participant who wore the prosthetic socket fabricated with the traditional handmade method due to its larger size; whereas, the interface stress in the tibia end was relatively high in both cases, while being more evident in case of the socket made using the RE and RP fabrication method.

### Satisfaction rating scale analysis results

**Fig. 15:**
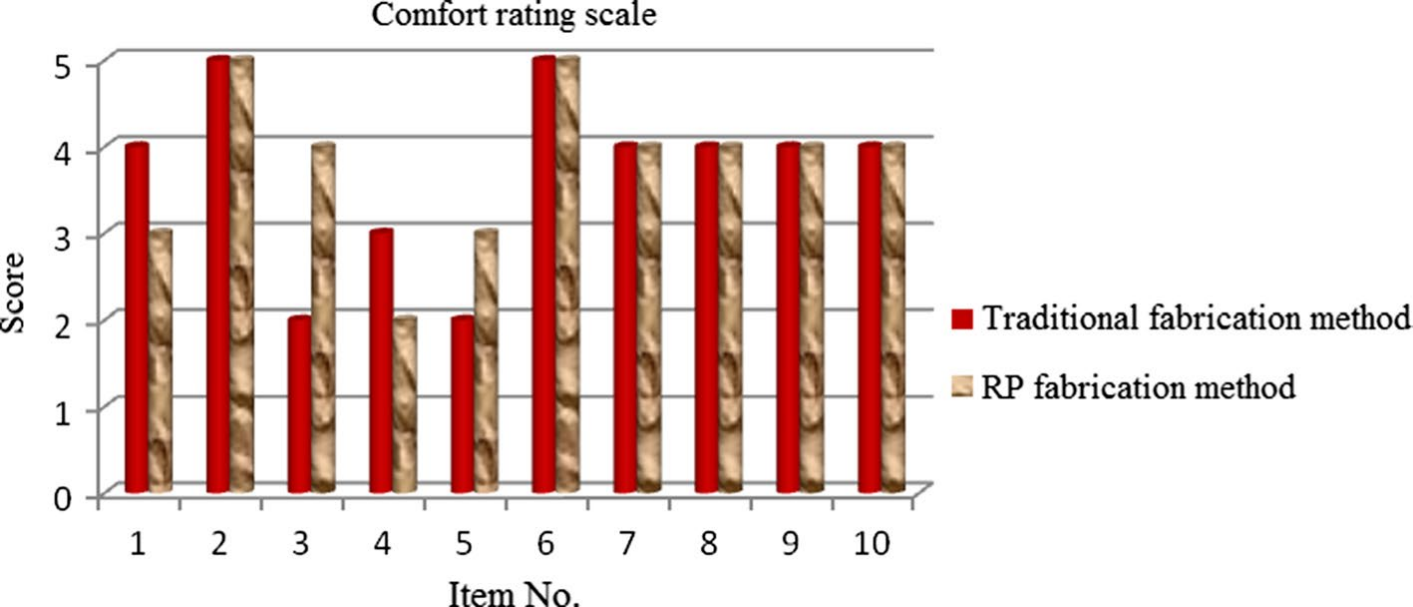
Comfort rating scale

**Fig. 16:**
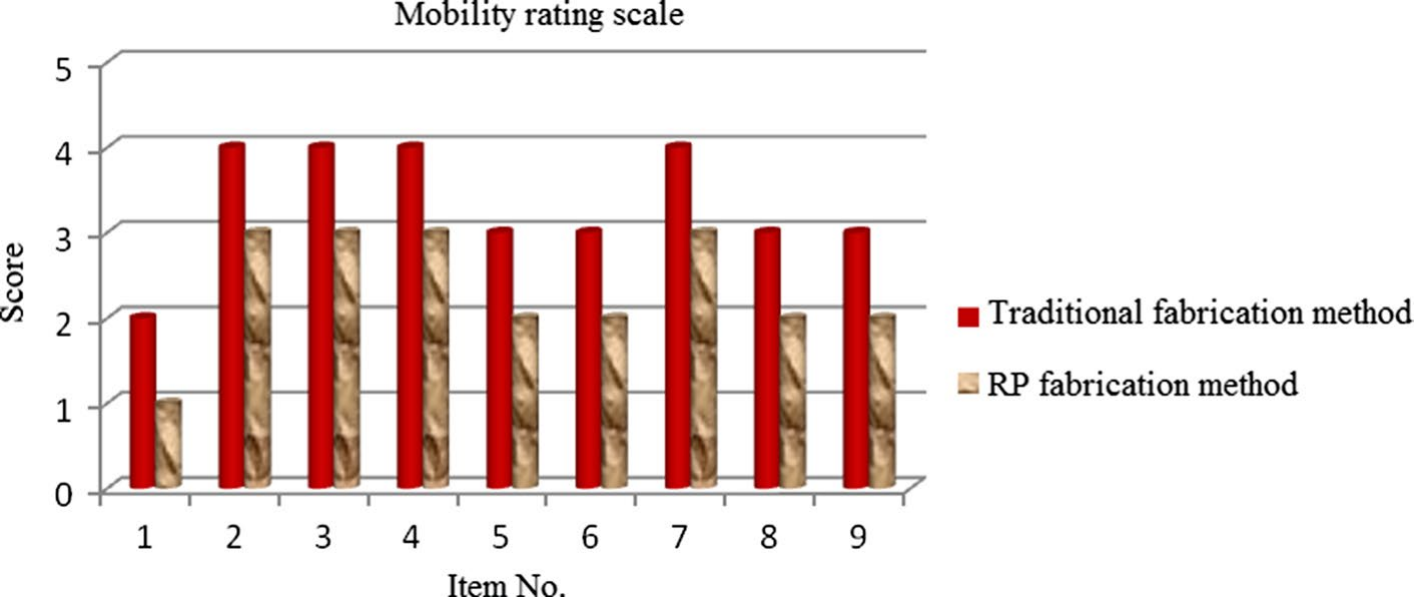
Mobility rating scale

The satisfaction rating scale was used to analyze amputees’ satisfaction with the prosthetic sockets fabricated with the traditional handmade method and RE and RP in terms of their comfort level and mobility. The comfort dimension included items regarding the ease of taking on and off the prosthesis and factors affecting the amputee’s subjective perception, such as aesthetic appearance, smell, and sound of the prosthesis (Table 6). Comfort-related satisfaction results are presented in Fig. 15. The mobility dimension was related to the participants’ walking and included stability when walking up or down stairs or slopes, convenience when getting in and out of vehicles, and the ability to walk on slippery surfaces or in narrow places (Table 7). Mobility-related satisfaction results are presented in Fig. 16. The results showed that, for comfort, the prosthetic sockets fabricated using RE and RP showed better results than those fabricated with the traditional handmade method in terms of prosthesis weight and the amount of required prosthetic socks; whereas, no significant difference was observed for other items. In the mobility dimension, the prosthetic sockets fabricated with the traditional handmade method outperformed those fabricated using RE and RP. The results showed that, at the time of the survey, the participants were more satisfied with the prosthetic sockets fabricated with the traditional handmade method.

**Table 6:** Comfort level dimension content

Item	Score	Subjective perception content	Patient’s subjective perception
The prosthesis feels comfortable when standing up and sitting down	1	Absolutely uncomfortable	
	2	A bit uncomfortable	
	3	Satisfactory	
	4	Comfortable	
	5	Very comfortable	
The prosthesis is easy to take on and off	1	Impossible	
	2	Very difficult	
	3	Moderately difficult	
	4	A bit difficult	
	5	Easy	
The prosthesis feels light	1	Very heavy	
	2	A bit heavy	
	3	Satisfactory	
	4	Light	
	5	Very light	
After wearing the prosthesis for how long does it feel hot?	1	1 h	
	2	1–4 h	
	3	4–7 h	
	4	7–11 h	
	5	12 h and more	
How many prosthetic socks are needed?	1	Silk socks × 1; silica gel socks × 1; thick quilted socks × 3	
	2	Silk socks × 1; silica gel socks × 1; thick quilted socks × 2	
	3	Silk socks × 1; silica gel socks × 1; thick quilted socks × 1	
	4	Silk socks × 1; silica gel socks × 1; thin quilted socks × 1	
	5	Silk socks × 1; thin quilted socks × 2	
The prosthesis makes weird sounds	1	Always	
	2	Frequently	
	3	Sometimes	
	4	Rarely	
	5	Never	
When on, the prosthesis causes an allergic reaction to skin	1	Always	
	2	Frequently	
	3	Sometimes	
	4	Rarely	
	5	Never	
It is possible to wear any type of shoes	1	Impossible	
	2	Usually possible	
	3	Sometimes possible	
	4	Usually possible	
	5	Possible	
The prosthesis is resistant to dirt and easy to clean	1	Impossible to clean	
	2	Usually impossible to clean	
	3	Sometimes possible to clean	
	4	Usually possible to clean	
	5	Possible to clean	
The prosthesis makes it difficult to dress	1	Always	
	2	Frequently	
	3	Sometimes	
	4	Rarely	
	5	Never

**Table 7:** Mobility dimension content

Item	Score	Subjective perception content	Patient’s subjective perception
How long can you walk while wearing the prosthesis?	1	Less than 1 h	
	2	1–4 h	
	3	4–7 h	
	4	7–11 h	
	5	More than 12 h	
Do you feel stable when walking on uneven surfaces while wearing the prosthesis?	1	Absolutely unstable	
	2	Very unstable	
	3	Moderately unstable	
	4	A bit unstable	
	5	Stable	
Is it difficult to walk fast with the prosthesis for half an hour?	1	Impossible	
	2	Very difficult	
	3	Moderately difficult	
	4	A bit difficult	
	5	Easy	
It is difficult to walk up and down stairs while wearing the prosthesis?	1	Impossible	
	2	Very difficult	
	3	A bit difficult	
	4	Able to keep pace with a healthy person	
	5	Walking as a healthy person	
How much physical strength is required to use the prosthesis?	1	Unable to walk	
	2	Requires much effort	
	3	Requires some effort	
	4	Requires little effort	
	5	Does not require any effort	
Can you walk stably up and down slopes while wearing the prosthesis?	1	Impossible	
	2	Very difficult	
	3	A bit difficult	
	4	Able to keep pace with a healthy person	
	5	Walking as a healthy person	
Can you get in and out of a vehicle while wearing the prosthesis?	1	Impossible	
	2	Very difficult	
	3	Moderately difficult	
	4	A bit difficult	
	5	Easy	
Can you walk on slippery surfaces while wearing the prosthesis?	1	Impossible	
	2	Very difficult	
	3	A bit difficult	
	4	Able to keep pace with a healthy person	
	5	Walking as a healthy person	
Can you walk stably on narrow streets while wearing the prosthesis?	1	Absolutely unstable	
	2	Very unstable	
	3	Moderately unstable	
	4	A bit unstable	
	5	Stable

## Conclusions

This study sought to compare amputees’ satisfaction with prosthetic sockets fabricated under different processing conditions, particularly, using RE and RP and the traditional handmade method. The main factors affecting their satisfaction with the prostheses were gait normality and stump–socket interface stress distribution. For the sake of accuracy, this study conducted an empirical analysis of gait and interface stress and measured the prosthesis users’ satisfaction levels. The following conclusions were made based on the results:

1. With regards to gait analysis, a greater difference in step length between the amputated and healthy limbs was observed in the participant who wore the prosthetic socket fabricated using RE and RP, meaning that asymmetry between two limbs is a serious issue in the use of such prosthetic sockets. Observation of the gait cycles found that the healthy limbs had greater stance phases than the amputated limb. This indicated that the participant tended to put a greater load on the healthy limb, while not willing to load the amputated limb, which was more evident in case of the prosthetic socket fabricated using RE and RP. Gait analysis data demonstrated that the prosthetic socket fabricated using the traditional handmade method had better characteristics.
2. With regards to stump–socket interface stress, this study found that, regardless of the method used for socket fabrication, most stress was concentrated in tibia end pressure-relief area. This caused discomfort in the area of tibia end to the participant wearing prosthesis. This discomfort was most evident in case when the prosthetic socket was fabricated using RE and RP.
3. Analysis of the survey data obtained with the satisfaction rating scale showed a higher level of satisfaction toward prosthetic sockets fabricated with the traditional handmade method rather than those fabricated using RE and RP. The latter outperformed the former in terms of comfort level, while being inferior in terms of user satisfaction towards the prosthesis mobility. These results imply that mobility is an important consideration when introducing new prosthetic sockets to an amputee.
4. Survey data analysis showed that important factors for prosthesis users included the sensation of heat, ease of taking on and off the prosthesis, and its weight and mobility. Another important issue is reducing back and lower back pain in prosthesis users. It is hoped that the survey results can serve as a reference for the design and fabrication of prosthetic sockets.

## Abbreviations

CAD: computer-aided design
CAE: computer-aided engineering
RE: reverse engineering
RP: rapid prototyping
NRS: numeric rating scale
VAS: visual analogue scale
CT: computed tomography
